# Supplementary material for: Influenza viral infection at the plasma membrane is restricted by lipid composition
Source: J Virol. 2025 Jul 24;99(8):e01105-25. doi: 10.1128/jvi.01105-25 (PMC12363186; doi:10.1128/jvi.01105-25)
Supplement: Supplemental figures — Figures S1 to S6. [file jvi.01105-25-s0001.pdf]

Supplementary Material for:

Influenza viral infection at the plasma membrane is restricted by lipid composition

Steinar Mannsverk, Ana M. Villamil Giraldo, and Peter M. Kasson

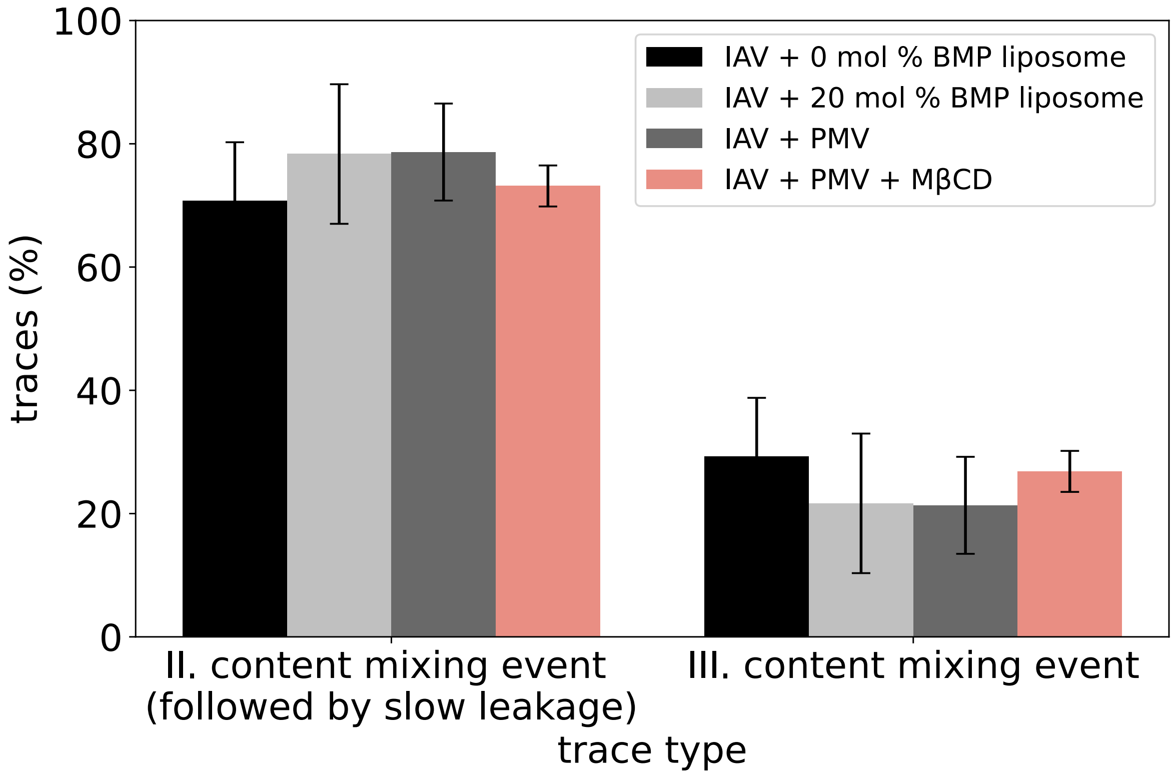

Figure S1. Distribution of IAV content mixing events classified as Type II or III. The % of traces which displayed the characteristic calcein intensity followed by a slow decay (Type II) or stable elevation (Type III). See **Fig. 3c** for sample traces for each type. Bars represent mean ± standard error mean for each target membrane, as indicated. No statistically significant difference between target membranes was found.

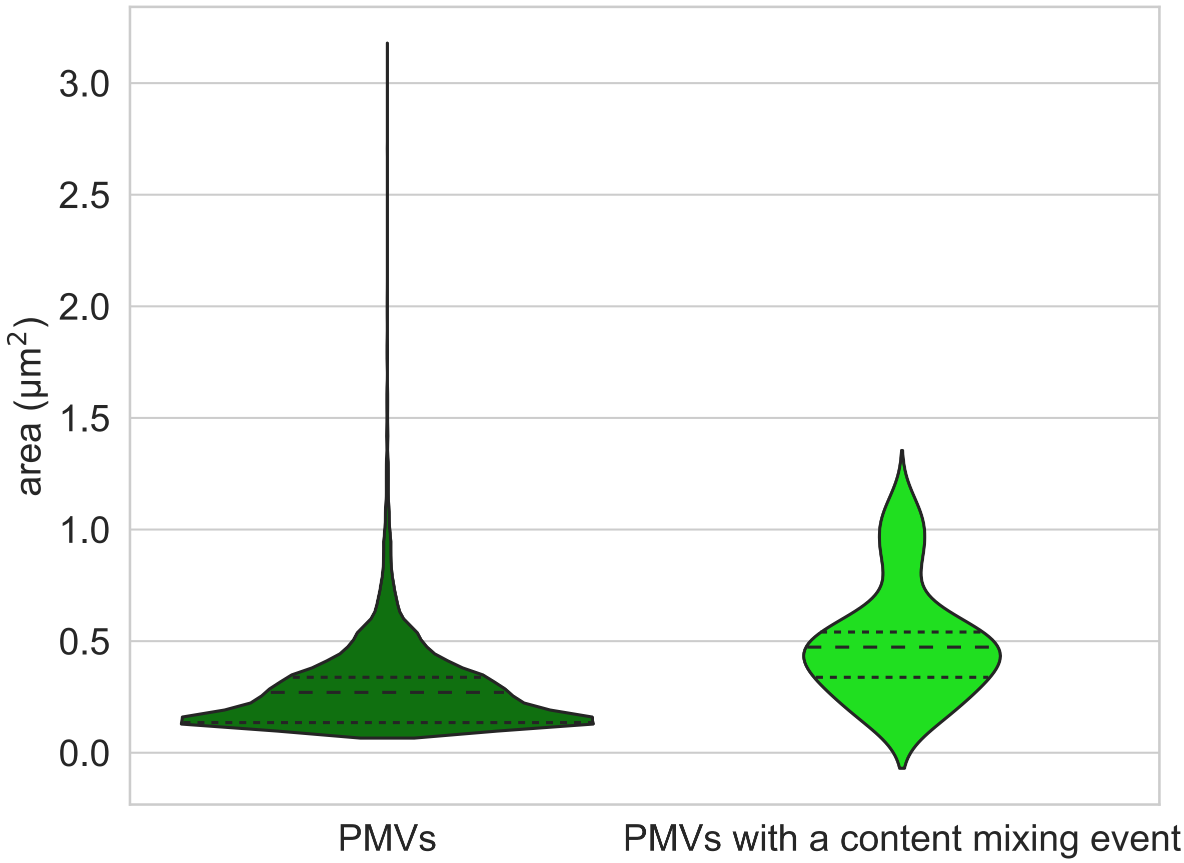

Figure S2. Size distribution of PMVs undergoing a content mixing event after IAV fusion triggering. The PMV particle size was measured using the ComDet (v.0.5.5) plugin in ImageJ, with the following settings: ch1a = 2 and ch1s = 3. Next the PMVs that underwent a content mixing event was paired with their respective size measurement from the ComDet analysis, through measuring the shortest euclidean distance (but ≤3 pixels away) between the two datasets, from their respective X and Y coordinates. Lastly, a Wilcoxon signed-rank test was performed to test whether the size distribution between the PMVs and PMVs that underwent a content mixing event was significantly different (p-value = 0.125).

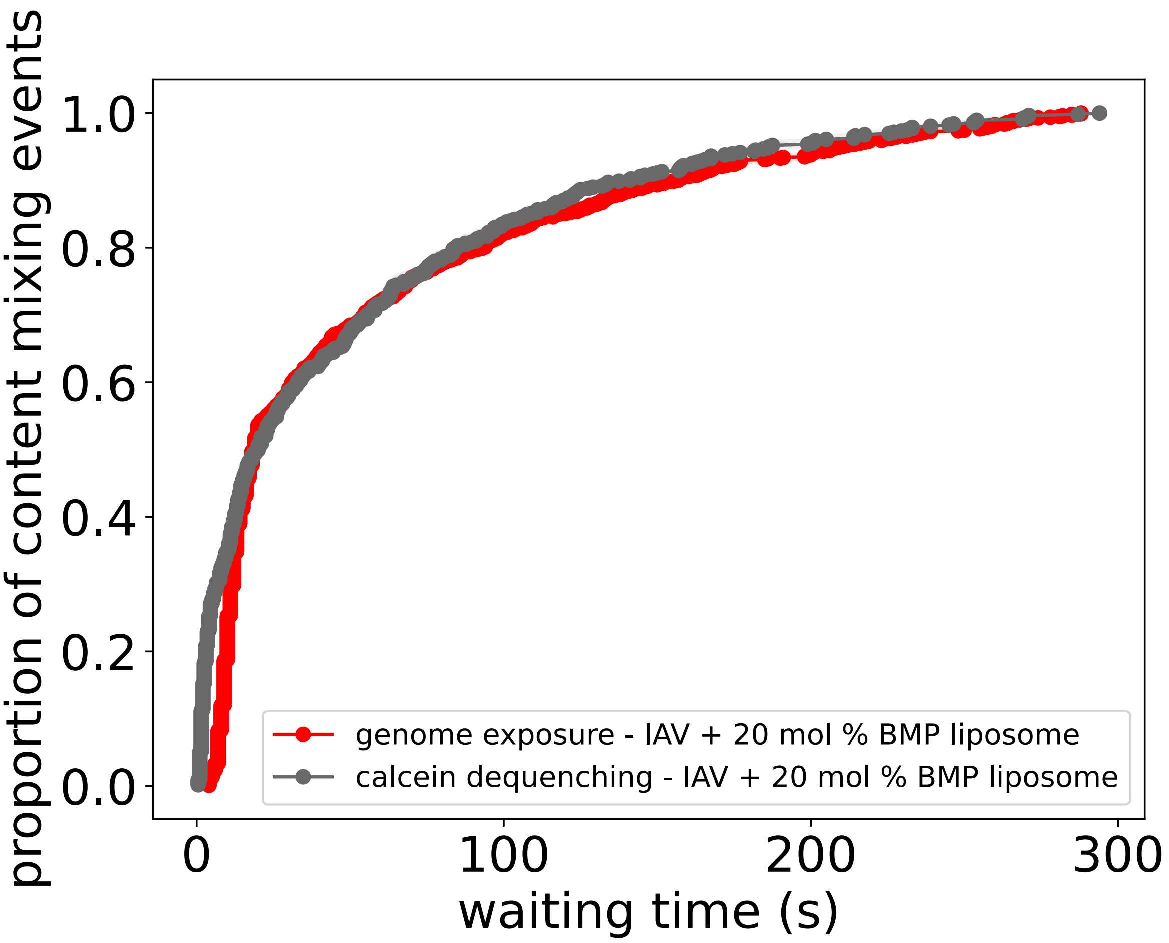

Figure S3. Comparison of content mixing kinetics between different fluorophores. The cumulative distribution functions (CDFs) of event waiting times for IAV bound to 20 mol % BMP liposomes loaded with a nucleic acid-binding dye (DiYO-1), described previously (1) or calcein, as described here. The genome exposure CDF is replotted from the prior data (1). Note that the genome exposure CDF is right-shifted by a few seconds due to the difference in how time 0 was determined: For the genome exposure assay, time 0 = when the buffer exchange is initiated, while for the calcein dequenching assay time 0 = when the background calcein signal drops due to the pH sensitivity of calcein. The latter is thought to be a more precise measure of when the viral particle is exposed to the low pH buffer.

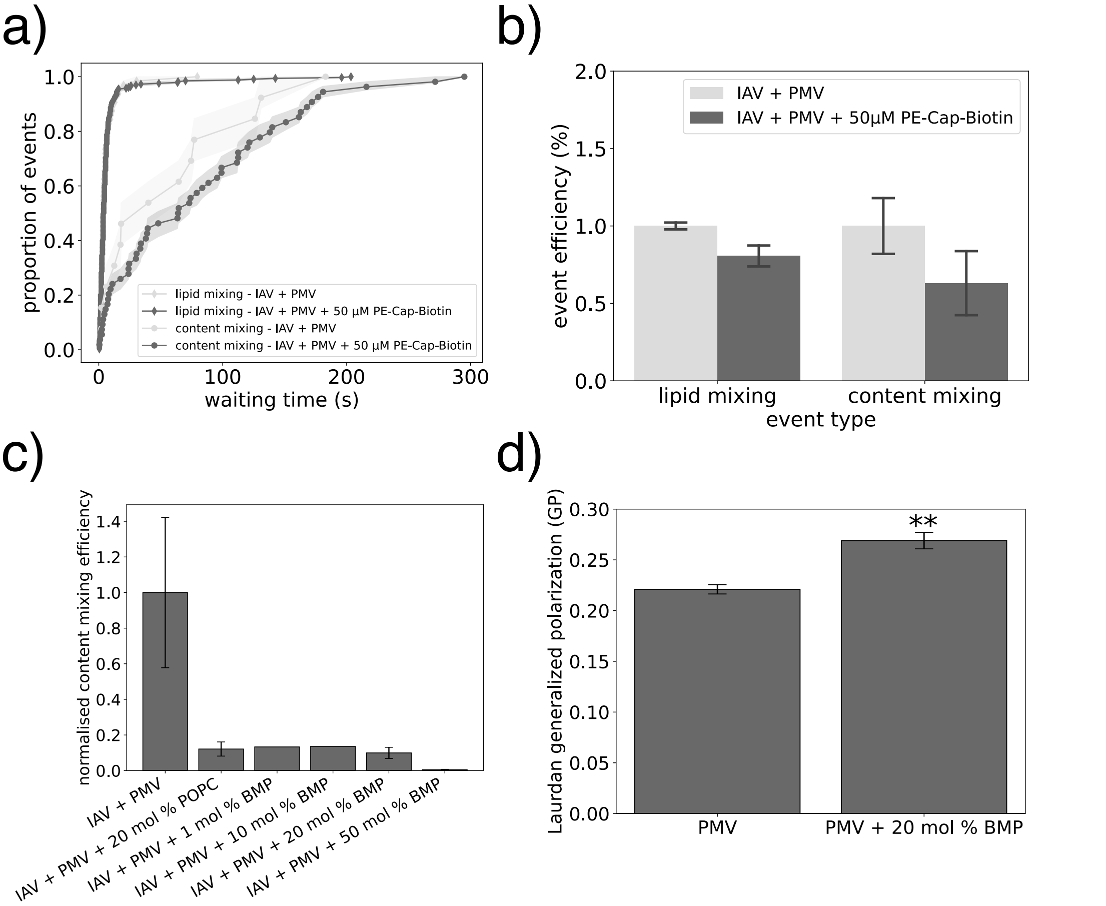

Figure S4. Content mixing kinetics, efficiency and Laurdan generalized polarization of PMVs directly supplemented with exogenous lipids. a) Lipid and content mixing kinetics at 37°C of IAV bound to PMVs supplemented with 50 μM PE-Cap-Biotin, shown as normalized cumulative distribution functions (CDFs) of event waiting times. Three independent channels were analyzed for each condition. Filled area around CDFs represent the bootstrapped interquartile range and statistical testing was performed as described in the Methods. **b**) Lipid and content mixing efficiency, calculated as total events recorded / TR-labeled IAV particles detected in the same field of view. Bars show mean ± standard error. Two separate two-sample t-tests showed no significant difference between lipid mixing (p-value = 0.091) or content mixing (p-value = 0.25) efficiency. **c**) Content mixing efficiency is plotted between IAV and PMVs supplemented with BMP or POPC. For these experiments, the exogenous lipid was dissolved in chloroform followed by shaking at 800 rpm at room temperature for 30 mintues to promote lipid incorporation. The amount of lipid to add to the PMV sample was estimated (see **Supplementary methods item 1**) and the volume of solvent added to the PMV sample was less than 1 % of the total sample volume. Bars show mean mixing efficiency, calculated as content mixing events / HA-positive particles in each field of view. Error bars represent standard error and are plotted for samples with at least 3 individual repeats. Values are normalised to the content mixing efficiency of untreated PMVs. **d**) Laurdan generalized polarization (GP) is plotted for PMVs supplemented with BMP directly prior to labelling with C-Laurdan. A one-way ANOVA and Tukey HSD post-hoc test was carried out to determine significant difference between groups. ** signifies a p-value < 0.01. See **Fig. 5b** for more information on Laurdan GP.

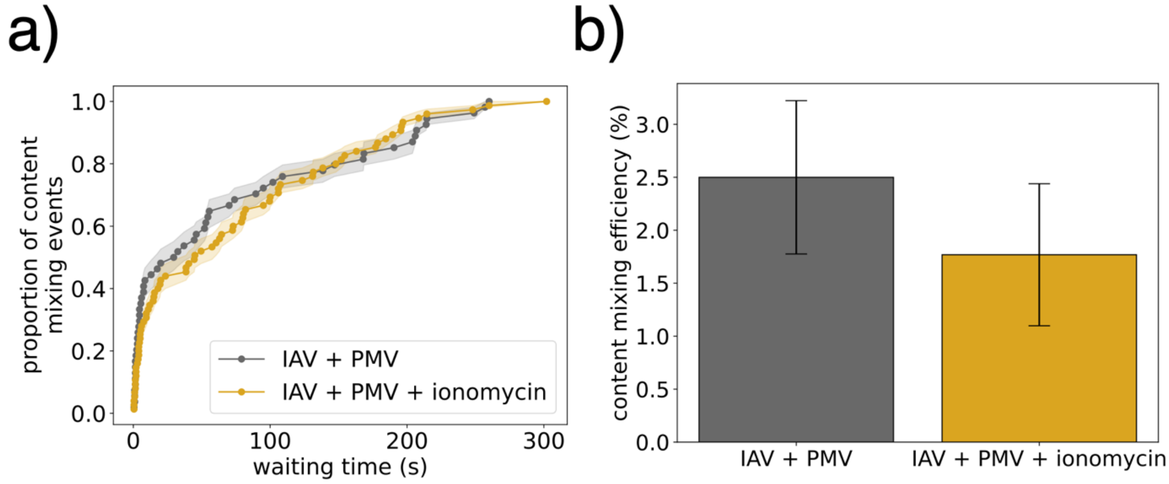

Figure S5. Content mixing kinetics and efficiency of IAV with lipid-scrambled PMVs. PMVs bound to a flow cell channel were pretreated with 10 μM ionomycin for 30 min at 37°C, before the ionomycin was washed out and IAV introduced. **a**) Kinetics are plotted as normalized CDFs of single-event waiting times. No statistically significant difference between the CDFs was found by a KS test (p-value = 0.58) or bootstrapped rank sum test over flow cells (p-value = 0.98). **b**) Content mixing efficiency is plotted, calculated as total events recorded / TR-labeled IAV particles detected in the same field of view. Bars show mean ± standard error. No significant difference between group was found using a two-sample t-test (p-value = 0.50)

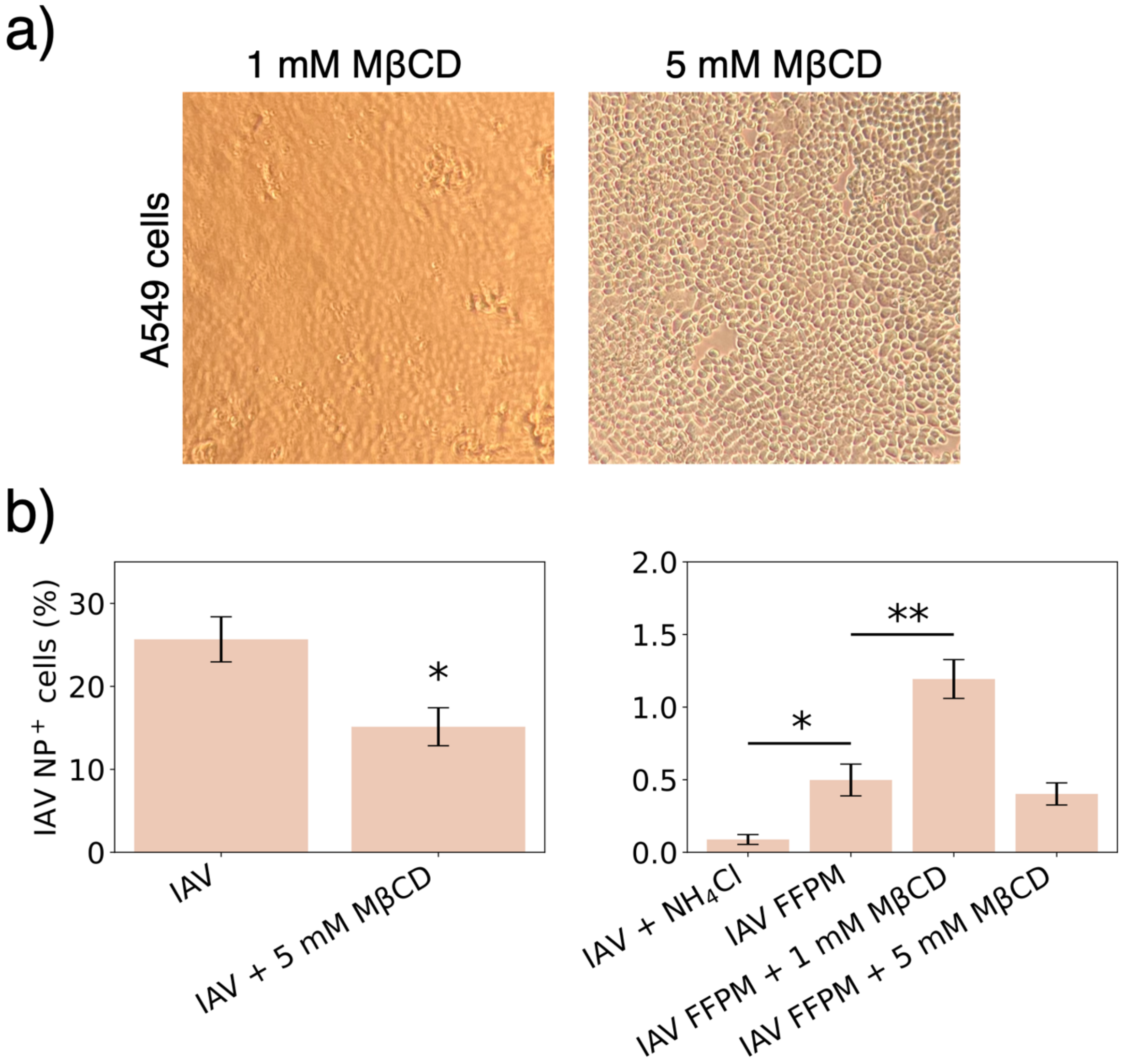

Figure S6. IAV FFPM in A549 cells pre-treated with MβCD. a) bright-field images of an A549 monolayer after treatment with 1 mM (left image) or 5 mM (right image) MβCD for 30 min at 37°C. Cells in the right image show loss of cell-cell adherence after MβCD treatment. However, the cells remained firmly attached to the bottom of the well throughout the FFPM infection experiment. **b**) Quantification of IAV NP-positive cells 5 hours post-infection. NP positivity was defined as a mean nuclear NP intensity above uninfected cells, and the percent NP-positive nuclei was calculated in each sample as in **Fig. 6b**. A one-way ANOVA and Tukey HSD post-hoc test was used to determine statistically significant differences between groups, with significantly different groups labelled on the plot. Bar plot shows mean of each group ± standard error mean from three repeats. * and ** indicate p-values < 0.05 and < 0.01, respectively. Note different y-axis range on left and right panel. However, experiments plotted in both panels were carried out in parallel and are therefore comparable.

References

1. Mannsverk S, Villamil Giraldo AM, Kasson PM. 2022. Influenza Virus Membrane Fusion Is Promoted by the Endosome-Resident Phospholipid

Bis(monoacylglycero)phosphate. The Journal of Physical Chemistry B 126:10445-10451.
