# Supplementary material for: Influenza viral infection at the plasma membrane is restricted by lipid composition
Source: J Virol. 2025 Jul 24;99(8):e01105-25. doi: 10.1128/jvi.01105-25 (PMC12363186; doi:10.1128/jvi.01105-25)
Supplement: Supplemental methods — Methods for estimating lipid mole fraction. [file jvi.01105-25-s0002.pdf]

To estimate the number of phospholipids in a single PMV, we first calculated the radii of the outer and inner leaflets of the vesicle membrane. The outer leaflet radius ($r_{outer\ leaflet}$) was taken as the mean vesicle radius, as given by the Nanosight nanoparticle tracker of the PMV sample. The inner leaflet radius ($r_{inner\ leaflet}$) was taken as r_{outer} minus half the assumed bilayer thickness (7 nm). Next, the area of the bilayer occupied by proteins was estimated to 25 %, hence the total lipid Bilayer area was adjusted accordingly (Dupuy and Engelman 2008):

$$\text{PMV bilayer area} = 4\pi(r_{\text{outer leaflet}})^2 + 4\pi(r_{\text{outer leaflet}} - 7\ \text{nm})^2 \times (1 - \text{protein content})$$

Next, the total number of phospholipids ($N_{PMV\ phospholipids}$) per PMV was estimated by dividing the PMV bilayer area with the assumed phospholipid area ($A_{phospholipid}$) of 0.513 nm^2, as reported elsewhere (Alberts et al. 2002):

$$N_{\text{PMV phospholipids}} = \frac{\text{PMV bilayer area}}{A_{\text{phospholipid}}}$$

To convert from estimated phospholipids per PMV to total phospholipids in the sample ($N_{sample\ phospholipids}$), the $N_{PMV\ phospholipids}$ was multiplied by the PMV concentration ($C_{PMV\ sample}$), as determined by the Nanosight nanoparticle tracker, and the total sample volume ($V_{PMV\ sample}$):

$$N_{\text{sample phospholipids}} = N_{\text{PMV phospholipids}} \times C_{\text{PMV sample}} \times V_{\text{PMV sample}}$$

Lastly, the amount of phospholipids to supplement the PMV to reach a desired mol % ratio of the total phospholipid content was estimated by:

$$N_{\text{phospholipid supplement}} = \frac{N_{\text{PMV phospholipids}} \times \text{ratio}_{\%\ \text{mol}}}{(1 - \text{ratio}_{\%\ \text{mol}})}$$

The volume of a certain phospholipid to add to the PMV sample to reach the desired mol % ratio was calculated by the molar weight of the phospholipid and molarity of the stock solution.

References:

Alberts, Bruce, Alexander Johnson, Julian Lewis, Martin Raff, Keith Roberts, and Peter Walter. 2002. ‘The Lipid Bilayer’. In *Molecular Biology of the Cell. 4th Edition*. Garland Science. https://www.ncbi.nlm.nih.gov/books/NBK26871/.

Dupuy, Allison D., and Donald M. Engelman. 2008. ‘Protein Area Occupancy at the Center of the Red Blood Cell Membrane’. *Proceedings of the National Academy of Sciences of the United States of America* 105 (8): 2848–52. https://doi.org/10.1073/pnas.0712379105.
